# Uncovering temperature sensitivity of West Nile virus transmission: Novel computational approaches to mosquito-pathogen trait responses

**DOI:** 10.1371/journal.pcbi.1012866

**Published:** 2025-03-31

**Authors:** Julian Heidecke, Jonas Wallin, Peter Fransson, Pratik Singh, Henrik Sjödin, Pascale Claire Stiles, Marina Treskova, Joacim Rocklöv

**Affiliations:** 1 Interdisciplinary Center for Scientific Computing, Heidelberg University, Heidelberg, Germany,; 2 Heidelberg Institute of Global Health, Heidelberg University, Heidelberg, Germany; 3 Department of statistics, Lund university, Lund, Sweden; 4 Department of public health and clinical medicine, Section of sustainable health, Umeå university, Umeå, Sweden; Wageningen UR: Wageningen University & Research, NETHERLANDS, KINGDOM OF THE

## Abstract

Temperature influences the transmission of mosquito-borne pathogens with significant implications for disease risk under climate change. Mathematical models of mosquito-borne infections rely on functions that capture mosquito-pathogen interactions in response to temperature to accurately estimate transmission dynamics. For deriving these functions, experimental studies provide valuable data on the temperature sensitivity of mosquito life-history traits and pathogen transmission. However, the scarcity of experimental data and inconsistencies in methodologies for analysing temperature responses across mosquito species, pathogens, and experiments present major challenges. Here, we introduce a new approach to address these challenges. We apply this framework to study the thermal biology of West Nile virus (WNV). We reviewed existing experimental studies, obtaining temperature responses for eight mosquito-pathogen traits across 15 mosquito species. Using these data, we employed Bayesian hierarchical models to estimate temperature response functions for each trait and their variation between species and experiments. We incorporated the resulting functions into mathematical models to estimate the temperature sensitivity of WNV transmission, focusing on six mosquito species of the genus *Culex*. Our study finds a general optimal transmission temperature around 24°C among *Culex* species with only small species-specific deviations. We demonstrate that differing mechanistic assumptions underlying published mosquito population models result in temperature optima estimates that differ by up to 3°C. Additionally, we find substantial variability between trait temperature responses across experiments on the same species, possibly indicating significant intra-species variation in trait performance. We identify mosquito biting rate, lifespan, and egg viability as priorities for future experiments, as they strongly influence estimates of temperature limits, optima, and overall uncertainty in transmission suitability. Experimental studies on vector competence traits are also essential, because limited data on these currently require model simplifications. These data would enhance the accuracy of our estimates, critical for anticipating future shifts in WNV risk under climate change.

## Author summary

Diseases transmitted by mosquitoes are sensitive to changes in environmental conditions. Ambient temperature directly impacts mosquitoes’ ability to survive, reproduce, and transmit pathogens. West Nile virus (WNV) is a pathogen transmitted almost globally and a major cause off mosquito-borne illnesses in temperate regions. In this study, we explore how temperature affects the transmission of WNV by six mosquito species. This knowledge is crucial for predicting the spread of mosquito-borne diseases, particularly in the light of climate change. We used a large dataset of laboratory experimental data and employed advanced statistical and mathematical models to analyze which temperatures are optimal for WNV transmission by these mosquitoes. Our findings reveal that the optimal temperature for WNV transmission is around 24°C. We, however, observe that different configurations of the mathematical transmission models cause variations in this optimal temperature, highlighting the importance of careful model selection. Additionally, we observe significant differences in how mosquitoes respond to temperature changes in different experiments, even within the same species. Lastly, our study emphasizes the need for more comprehensive experimental data on specific mosquito species to improve predictions of WNV suitability in regions where these species are relevant vectors of WNV.

## Introduction

West Nile virus (WNV) is a pathogenic multi-host and multi-vector flavivirus. Transmission of the virus is maintained in an enzootic cycle between various wild birds and mosquitoes, primarily of the genus *Culex* (*Cx.*) [1,2]. Due to their opportunistic feeding behaviour, many *Culex* mosquitoes can act as bridge vectors by transmitting the virus from its enzootic cycle to humans, equids, and other mammals [3–5]. The latter are dead-end hosts which cannot transmit the virus back to mosquitoes but can be infected. In humans, approximately 25% of infections progress to West Nile fever, while less than 1% result in a neuroinvasive disease with a case fatality ratio of 10% [1]. Several vaccines have been licensed for use in horses, but there are currently no vaccines nor therapeutic drugs available for use in humans [6,7]. Historically, WNV was associated with outbreaks across Africa, Eurasia, Australia, and the Middle East [8]. In 1999, WNV was introduced to the United States, initially in New York City. There, the virus found a conducive environment and quickly spread across the United States from coast to coast within four years. WNV has since been introduced in other countries across the Americas and can now be found almost globally [9]. WNV has also been extending its geographic range in Europe since the late 1990s, although much more slowly than in the United States. However, major outbreaks in 2010 and 2018 marked unprecedented large transmission events with significant geographical expansion to previously unaffected areas. For example, the 2018 outbreak in Europe was characterized by 2,083 locally reported human cases, a 7.2-fold increase compared to the previous season [10].

Climate change alters the global landscape in which WNV and other mosquito-borne diseases (MBDs) manifest, creating more suitable conditions for mosquitoes to transmit diseases in temperate regions [11]. In fact, the expansion and increasing frequency of outbreaks of WNV in Europe appears to be driven by climate change [12]. Overall, there is ample evidence that climate change has amplified and is projected to continue amplifying WNV risk in several areas across the globe [11,13–17]. While climate change is only one of several drivers, along with land use, socio-economic conditions, and host mobility [17], suitable climatic conditions are necessary for the establishment and local transmission of any MBD [18]. Therefore, understanding climate-driven changes in WNV risk and having the tools to project such changes is essential for proactive adaptation to climate change.

Temperature is one of the key climatic drivers often limiting mosquito-borne disease risk in temperate regions [18,19]. Since mosquitoes are ectothermic organisms, temperature directly impacts their life-history traits such as lifespan, biting activity, and reproductive success. Furthermore, temperature impacts pathogen dynamics within mosquitoes, which affects the physical ability of mosquitoes to transmit pathogens and the extrinsic incubation period [19]. An extensive body of laboratory-based studies reports reactions of mosquito-pathogen traits to changes in temperature. Mechanistic models of MBDs based on such data predict that transmission suitability responds to temperature in a nonlinear, unimodal fashion with optimal temperatures depending on the mosquito species and pathogen combination [19–23]. Recent predictions for WNV transmission hint towards optimal temperatures around 22–25°C [20,24–26].

Several studies have attempted to compare how the temperature suitability for WNV varies across *Culex* species [15,20,26–28]. These comparisons are challenging due to the scarcity of laboratory experimental data. One strategy to address this is to partially pool data between species to regularize model fits of species with sparse data. However, previous studies that approached partial pooling for trait temperature response fits (see study [20] for WNV and [22,29] for other MBDs), have lacked a fully Bayesian approach for uncertainty quantification and comparison of species-specific estimates. Moreover, these previous studies have purely focused on the variability of temperature responses between species and pooled data across experiments on the same species. Overall, studies to date have disregarded the statistical dependencies introduced by experiment identity which can bias temperature response estimates and uncertainty quantification.

We address these gaps by introducing a formal statistical approach based on Bayesian hierarchical models. In this framework, hierarchical priors, estimated during model fitting, quantify the variability of temperature responses across species and experiments on the same species. This decomposition of variability accounts for data dependencies at the species- and experiment-level, leading to more accurate estimates and uncertainty quantification. Additionally, by using hierarchical priors, our approach provides a purely data-driven method for partial pooling and enables uncertainty quantification of the trait temperature responses for species with no data.

We applied this approach to an updated and expanded dataset of laboratory experimental studies that tested the temperature response of mosquito-pathogen traits [20]. Based on the new dataset, we derived temperature response fits for eight mosquito-pathogen traits across 15 mosquito species. Focusing on six *Culex* species (*Cx. pipiens, Cx. quinquefasciatus, Cx. pipiens molestus, Cx. pipiens pallens, Cx. restuans, Cx. tarsalis*) and their ability to transmit WNV, we derived species-specific estimates of the temperature response of WNV transmission suitability. Our transmission suitability model advances previous analyses by explicitly linking the model to mosquito population dynamics [20,25,26,28]. We further investigated how the choice of the mosquito population model influences the temperature optimum for WNV transmission and which trait estimates are important for temperature limits, optima, and overall uncertainty in transmission suitability.

From our analysis, we derive recommendations for MBD thermal biology analyses and identify critical data needs that would further strengthen the presented approach and enhance our understanding of climate change impacts on WNV and MBDs in general.

## Methods

**Fig 1:**
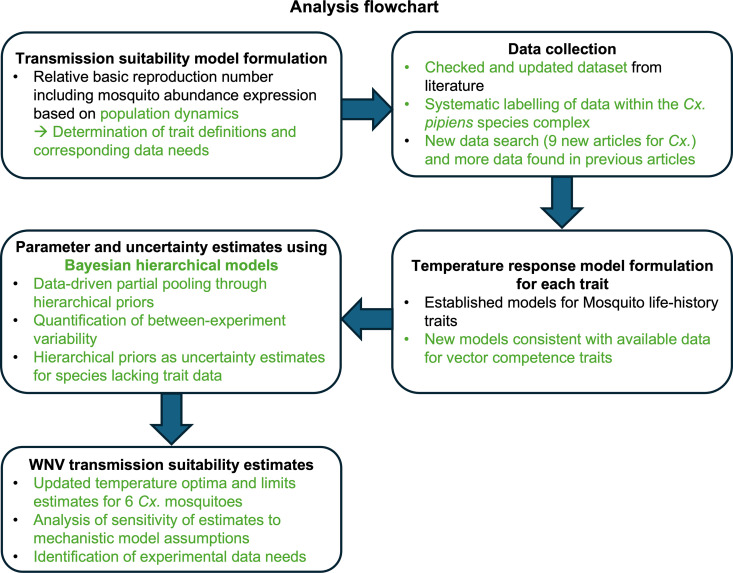
Graphical representation of our analysis. Key novelties in our appraoch compared to previous analyses of MBD thermal biology are highlighted in green.

We first introduce the mathematical model for WNV transmission suitability using a trait-based approach. Then we describe the process that we followed to derive a species-specific parameterization of this model. To this end, we proceed by outlining the data collection strategy that we used to update and extend a previously published dataset compiling outcomes of laboratory experiments that measured species’ trait performance at different temperatures [20]. We then introduce the functions that we fitted to these data to describe the temperature response of each mosquito-pathogen trait. A description of the Bayesian hierarchical modelling approach and the Markov Chain Monte Carlo implementation that we used to fit these functions follows. Finally, we describe how we propagated uncertainty from the trait temperature response fits to transmission suitability and the sensitivity and uncertainty analyses that we used to understand the contribution of each trait estimate to temperature limits, optima, and overall uncertainty in transmission suitability. Our analysis and its added value to previous analyses of MBD thermal biology of WNV [20,25,26] and other pathogens [19,22,23,29–32] are summarized in Fig 1.

### Mathematical models of WNV transmission suitability

**Fig 2:**
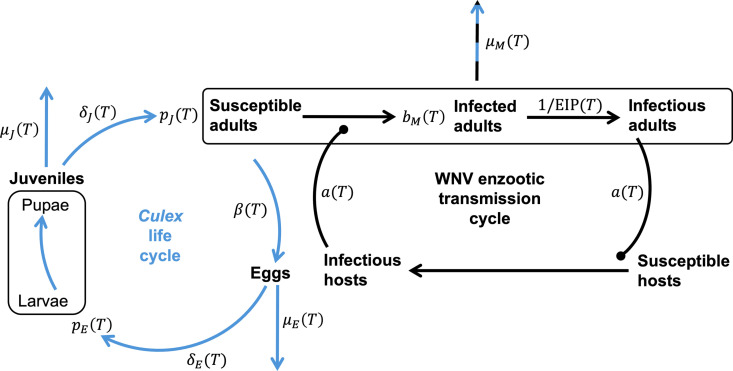
*Culex* life cycle (blue) and WNV transmission cycle (black) as well as the temperature-dependent parameters involved in both processes. Pointed arrows represent stage transitions and blunted arrows represent contact events facilitating disease transmission. Parameters to the side of the arrows represent rates, while parameters at the end of the arrows represent transition probabilites. Temperature-independent parameters and processes are left out of the figure. A detailed description of the temperature-dependent parameters can be found in Table 1.

The basic reproduction number $R_{0}$ , i.e., the expected number of secondary infections produced by a single infected host in a completely susceptible population (and related variables such as vectorial capacity) is arguably one of the most important metrics in infectious disease epidemiology and has been used in the past to capture the effect of temperature on different mosquito-borne diseases [19,21]. Here, we modelled the impact of temperature on transmission suitability by considering a relative version of the basic reproduction number. Fig 2 illustrates the WNV transmission cycle and *Culex* mosquito life cycle underlying our models, including the temperature-dependent parameters influencing both processes.

**Table 1:** **Description of the temperature-dependent parameters appearing in** $R_{0}^{\text{rel}}\left(T\right)$ **and the mosquito population dynamics model.**

Parameter (unit)	Description	Data type used for fitting/Calculation
$a\left(T\right)$ (1/time)	Adult mosquito biting rate	The inverse of the gonotrophic cycle duration as measured from blood meal ingestion to egg laying
$\text{lf}\left(T\right)$ (time)	Adult mosquito lifespan	Adult mosquito lifespan observations
$\mu_{M}\left(T\right)$ (1/time)	Adult mosquito mortality rate	Derived via $\mu_{M}\left(T\right)=1/\text{lf}\left(\text{T}\right)$
$\delta_{J}\left(T\right)$ (1/time)	Juvenile mosquito development rate	The inverse of the time to develop from egg hatch to adult emergence (encompassing the larva and pupa stage)
$p_{J}\left(T\right)$ (dimensionless)	Probability of surviving larva and pupa development stage	The percentage of juveniles surviving from egg hatch to adult emergence (encompassing the larva and pupa stage)
$\mu_{J}\left(T\right)$ (1/time)	Juvenile mosquito mortality rate	Can be approximated via $p_{J}\left(T\right)=\frac{\delta_{J}\left(T\right)}{\delta_{J}\left(T\right)+\mu_{I}\left(T\right)}$
$\delta_{E}\left(T\right)$ (1/time)	Egg hatching rate	The inverse of the time from egg laying to larva emergence
$p_{E}\left(T\right)$ (dimensionless)	Egg viability: probability that egg hatches	The percentage off eggs hatching
$\mu_{E}\left(T\right)$ (1/time)	Egg mortality rate	Can be approximated via $p_{E}\left(T\right)=\frac{\delta_{E}\left(T\right)}{\delta_{E}\left(T\right)+\mu_{E}\left(T\right)}$
$\beta_{\text{ER}}$ (dimensionless)	Number of eggs per egg raft	Egg count per egg raft
$\beta \left(T\right)$ (1/time)	Female mosquito egg laying rate	Derived via $\beta \left(T\right)=a\left(T\right)\beta_{\text{ER}}$
$b_{M}\left(T\right)$ (dimensionless)	Mosquito infection probability: probability of mosquitoes to develop a midgut infection after exposure to infected blood	Data from WNV vector competence studies testing mosquitoes for virus in their bodies after exposure to infected blood
$\text{EIP}\left(T\right)$ (time)	Extrinsic incubation period: average time required for infected mosquitoes to develop a salivary gland infection after exposure to infected blood	Data from WNV vector competence studies testing mosquitoes for virus in their salivary glands at different time points after exposure to infected blood

We started by considering a classical Ross-Macdonald equation [33,34] that models $R_{0}$ as given by


$$\begin{array}{c} R_{0}\left(T\right)=\frac{m\left(T\right)a{\left(T\right)}^{2}b_{M}\left(T\right)b_{H}e^{-\mu_{M}\left(T\right)\text{EIP}\left(T\right)}}{\mu_{M}\left(T\right)r_{H}} \end{array}$$ (1)

which incorporates the following temperature $\left(T\right)$ -dependent mosquito-pathogen traits:

- Adult mosquito biting rate $a\left(T\right)$
- Adult mosquito mortality rate $\mu_{M}\left(T\right)$
- Mosquito infection probability $b_{M}\left(T\right)$ (the probability of mosquitoes to develop a midgut infection after exposure to infected blood)
- Extrinsic incubation period $\text{EIP}\left(T\right)$ (average time required for infected mosquitoes to develop a salivary gland infection after exposure to infected blood)
- Mosquito to host ratio $m\left(T\right)=M\left(T\right)/H$

Additionally, the $R_{0}$ model includes the temperature-independent host competence $b_{H}$ and host recovery rate $r_{H}$. The model could be further extended by other temperature-independent traits such as host preference [25]. However, instead of focusing on the classical interpretation of $R_{0}$ as a threshold quantity we followed previous studies [19,20,25,26] and considered a relative version of $R_{0}$ that isolates the impact of temperature via the nonlinear interaction of mosquito-pathogen traits. To this end, we removed the temperature-independent host competence $b_{h}$, host recovery rate $r_{h}$, and host density *H* from Equation (1), which results in the following relative version of $R_{0}$:


$$\begin{array}{c} R_{0}^{\text{rel}}\left(T\right)=\frac{M\left(T\right)a{\left(T\right)}^{2}b_{M}\left(T\right)e^{-\mu_{M}\left(T\right)\text{EIP}\left(T\right)}}{\mu_{M}\left(T\right)} \end{array}$$ (2)

We normalised the resulting metric $R_{0}^{\text{rel}}\left(T\right)$ to $\left[0,1\right]$ by dividing by the maximum obtained value with respect to temperature.

To capture the impact of temperature on mosquito abundance $M\left(T\right)$, we considered a simple stage-structured mosquito population dynamics model:


$$\begin{array}{l} \dot{E}=\beta \left(T\right)M-\delta_{E}\left(T\right)E-\mu_{E}\left(T\right)E\\ \begin{array}{c} \begin{array}{l} \dot{J}=\delta_{E}\left(T\right)E-\alpha J^{2}-\delta_{J}\left(T\right)J-\mu_{J}\left(T\right)\\ \dot{M}=\omega \delta_{J}\left(T\right)J-\mu_{M}\left(T\right)M \end{array} \end{array} \end{array}$$ (3)

The model describes the dynamics across mosquito eggs *E*, juveniles *J* (encompassing larvae and pupae), and female adults *M* and depends on the following additional temperature-dependent mosquito life-history traits:

- Adult mosquito egg laying rate $\beta \left(T\right)$
- Egg hatching rate $\delta_{E}\left(T\right)$
- Egg mortality rate $\mu_{E}\left(T\right)$
- Juvenile mosquito development rate $\delta_{J}\left(T\right)$
- Juvenile mortality rate $\mu_{J}\left(T\right)$

The egg hatching and juvenile development rate in relation to the egg and juvenile mortality rate relate to egg viability (the percentage of eggs hatching) $p_{E}\left(T\right)$ and juvenile survival from egg hatch to adult emergence $p_{J}\left(T\right)$ via:


$$\begin{array}{c} p_{X}\left(T\right)=\frac{\delta_{X}\left(T\right)}{\delta_{X}\left(T\right)+\mu_{X}\left(T\right)},X\in \left\{E,J\right\}. \end{array}$$ (4)

We further defined $p_{EJ}\left(T\right)=p_{E}\left(T\right)p_{J}\left(T\right)$ as the probability of surviving both the egg and the juvenile stage.

The decision to separate the aquatic stage into two stages represented by eggs and juveniles (larvae and pupae combined) was driven by data availability. A more detailed separation of the different stages would need experiments that report trait data for larvae separate from pupae or even on different larval instar stages [35,36]. However, such a model would not have allowed using the large body of experiments that report development times and survival only on the combined larval and pupal stages [26,37–50] (see also Table J in S1 Text). The parameter ω represents the proportion of female mosquitoes at adult emergence and was assumed to be 0.5. Furthermore, the model incorporates a competition-driven juvenile mosquito mortality controlled by the parameter *α*. Competition in the juvenile aquatic stages is a common phenomenon leading to increased mortality and prolonged development times as well as carry-over effects on adult traits [41–43,51,52]. Here we incorporated the effect of increased competition-driven mortality at high larval density using a quadratic term $-\alpha J^{2}$ which assumes pair-wise individual interactions driving competition [53,54]. In the above model, this mechanism prevents unlimited mosquito population growth.

We used the adult female demographic equilibrium of the dynamic model (3) given by


$$\begin{array}{c} M^{*}\left(T\right)=\frac{1}{\alpha }\frac{\omega^{2}\beta \left(T\right)p_{E}\left(T\right)\delta_{J}{\left(T\right)}^{2}}{\mu_{M}{\left(T\right)}^{2}}\left[1-\frac{\mu_{M}\left(T\right)}{\omega \beta \left(T\right)p_{EJ}\left(T\right)}\right] \end{array}$$ (5)

as a proxy for mosquito abundance in the $R_{0}^{\text{rel}}\left(T\right)$ model (2) whereby $M^{*}\left(T\right)=0$ if the population reproduction number


$$\begin{array}{c} Q_{0}\left(T\right)=\frac{\omega \beta \left(T\right)p_{EJ}\left(T\right)}{\mu_{M}\left(T\right)} \end{array}$$ (6)

is less than or equal to one. The realized value of *α* depends on the availability of suitable space for juvenile development which we did not further consider in this study. At the demographic equilibrium, the factor 1/α is simply a scaling factor that we assumed to be temperature independent. Therefore, it cancels out when deriving the normalised $R_{0}^{\text{rel}}\left(T\right)$ which is thus invariant to changes in *α*.

The $R_{0}^{\text{rel}}\left(T\right)$ metric (2) describes a temperature-dependent transmission risk space that predicts a temperature at which WNV transmission would be optimized $T_{\text{opt}}^{R}$ (i.e., where $R_{0}^{\text{rel}}\left(T\right)$ reaches one) and approximates lower and upper temperature limits $T_{\text{min}}^{R}$ and $T_{\text{max}}^{R}$ (i.e., where $R_{0}^{\text{rel}}\left(T\right)$ becomes zero). Since we modelled the mosquito infection probability $b_{M}\left(T\right)$ as strictly positive (see following sections), it is straightforward to see that $R_{0}^{\text{rel}}\left(T\right)$ is zero if and only if $M^{*}\left(T\right)$ is zero. Therefore, these temperature limits can be interpreted as limits for the stability of active mosquito populations. If temperatures stay outside these limits for an extended period, active mosquito populations, and thus transmission cycles, are unlikely to be sustained. The temperature interval that would support disease transmission is located within these temperature limits but depends not only on $R_{0}^{\text{rel}}\left(T\right)$ being larger than zero but on the absolute $R_{0}$ (1) being greater than one. This cannot be predicted from $R_{0}^{\text{rel}}\left(T\right)$ alone because it depends on additional location-specific, temperature-independent factors such as host population density and susceptibility.

The mosquito population dynamics model (3) underlying the adult mosquito equilibrium expression (5) makes specific assumptions on mosquito ecology. To test if our $R_{0}^{\text{rel}}\left(T\right)$ estimates might be affected by the mosquito model selection, we considered alternative formulations from the literature that differ from system (3) in their incorporation of intra-specific competition into the mosquito population dynamic model and thus for integrating the impact of temperature on the mosquito-to-host ratio [55–60]. We also considered an expression that lacks a rigorous link to mosquito population dynamics but has been used in a series of previous analyses of MBD thermal biology [19,20,22,23,25,26,28–30,32,61]. The equations for these different models can be found in section SI7 in S1 Text, where we also discuss their theoretical foundation.

### Data collection

An overview of the temperature-dependent mosquito-pathogen traits appearing in $R_{0}^{\text{rel}}\left(T\right)$ (2) and the mosquito population dynamics model (3) can be found in Table 1. This table also includes a description of the data type used to fit the temperature response of each trait or how the trait is derived from other traits.

The starting point of our data collection was a previously published dataset [20], which compiled outcomes of experimental studies that documented the temperature dependence of multiple mosquito-pathogen traits. This dataset primarily focused on viruses causing MBDs in temperate areas and data on mosquito species capable of transmitting these pathogens. Many of these species belong to the genus *Culex,* but the dataset also contains information about temperate species from the genera *Aedes* and *Culiseta*. Here, we provide an updated and enriched version of this dataset. As a first step to gathering the updated dataset, we double-checked each entry in the original dataset [20] with the content of the primary articles. This screening led to several changes to the dataset, including the correction of typos and the application of conceptional differences when handling the data. A detailed description of these changes can be found in section SI8 in S1 Text. Then we extended this dataset by identifying additional experimental studies on *Culex* species. We did this by combining a database search with the screening of references from review articles and other sources. Following the inclusion criteria established for collecting the initial dataset [20], we only incorporated results from experiments that assessed trait performance across a minimum of three distinct constant temperature settings (with one exception). In the case of vector competence studies, we introduced an additional restriction to studies that tested mosquitoes for at least three different time points post exposure to the virus. Details on our literature search can be found in section SI9 in S1 Text. In total, this search identified 9 additional studies [26,35,37,51,62–66].

The final dataset entails data from 40 experimental studies on 8 different mosquito-pathogen traits across 15 mosquito species [26,35–51,62–83]. An overview of the studies included in our final data collection can be found in Table J in S1 Text.

In our analysis, we focused on six *Culex* species (*Cx. pipiens, Cx. quinquefasciatus, Cx. pipiens molestus, Cx. pipiens pallens, Cx. restuans, Cx. tarsalis*) and their potential to transmit WNV. Nevertheless, we kept life-history information on the other temperate *Culex, Aedes,* and *Culiseta* species in the dataset. This additional data helped to derive hierarchical priors during model fitting and as a side product of our models, we provide updated trait temperature response estimates for these species as well. To note, our use of the classification “species” is taxonomically inaccurate and was applied for simplicity. In fact, the mosquito *Cx. pipiens pallens* represents a *Cx. pipiens* x *Cx. quinquefasciatus* subspecies and *Cx. pipiens molestus* an ecotype of *Cx. pipiens* (for more details, see section SI6 in S1 Text).

### Modelling the temperature-response of mosquito-pathogen traits

For each mosquito-pathogen trait we chose a suitable parametric function by visual inspection of the data and by drawing on prior studies and theories in mosquito thermal biology [19,20,23,84]. Below, we define the functions used for fitting these different traits. A more detailed reasoning for our modelling choices also highlighting important differences in our approach to earlier studies [20,22,23,25,26,29–31] can be found in section SI1 in S1 Text.

Guided by the metabolic theory of ecology [19,84,85], we modelled the juvenile mosquito development rate $\delta_{J}\left(T\right)$, egg hatching rate $\delta_{E}\left(T\right)$, and adult biting rate $a\left(T\right)$ to exhibit left-skewed unimodal responses to temperature. Specifically, we used a modified Briére function to describe these rates [86]:


$$\begin{array}{c} f^{B}\left(T;q,T_{\text{min}},T_{\text{max}}\right)= \{\begin{array}{rr} \frac{q}{c}T\left(T-T_{\text{min}}\right)\sqrt{T_{\text{max}}-T}, & \text{max}\left(0,T_{\text{min}}\right)<T<T_{\text{max}}\\ 0, & \text{else} \end{array} \end{array}$$ (7)

The parameters $q,T_{\text{min}},T_{\text{max}}$ are the estimation targets whereas *c* represents a constant that acts to scale *q* on a similar scale as $T_{\text{min}}$ and $T_{\text{max}}$. This helped to improve the speed and stability of the fitting procedure. In addition, we set the scaling factor such that the parameter *q* obtains similar values between the three traits, making the estimates of *q* and their variability between species and experiments comparable for these traits.

To describe the symmetrical unimodal response of juvenile survival $p_{J}\left(T\right)$ and egg viability $p_{E}\left(T\right)$ observed in our data and in previous works [19], we fitted a modified quadratic function for these traits:


$$\begin{array}{c} f^{Q}\left(T;q,T_{\text{min}},T_{\text{max}}\right)= \{\begin{array}{rr} \text{min}\left(\frac{q}{c}\left(T-T_{\text{min}}\right)\left(T_{\text{max}}-T\right),1\right), & T_{\text{min}}<T<T_{\text{max}}\\ 0, & \text{else} \end{array} \end{array}$$ (8)

Here again, we incorporated the constant *c* to bring the parameter *q* on a similar scale as $T_{\text{min}}$ and $T_{\text{max}}$.

For adult mosquito lifespan $\text{lf}\left(T\right)$, we followed previous studies [20,26] and modelled it by a linearly decreasing function truncated at zero:


$$\begin{array}{c} f^{L}\left(T;\alpha ,\beta \right)= \{\begin{array}{rr} -\beta T+\alpha , & T<\frac{\alpha }{\beta }\\ 0, & \text{else} \end{array} \end{array}$$ (9)

We estimated the parameters *β* and $T_{\text{max}}:=\alpha /\beta$ since we found it more intuitive to define priors for the transformed parameter $T_{\text{max}}$ than for the intercept *α*. Although the temperature response of this trait is most likely unimodal, we fitted a linear model since the shape of its response at low temperatures was neither indicated by the available data nor clearly defined by biological theory, as the mosquitoes considered here employ different forms of dormancy. As a conservative approach to trait performance at low temperatures, we plateaued the linear function after model fitting at the lowest observed temperature point in the dataset across all species (14°C).

We derived the female mosquito egg-laying rate $\beta \left(T\right)$ by multiplying the biting rate $a\left(T\right)$ with the number of eggs per egg raft $\beta_{\text{ER}}$. The data available for eggs per egg raft $\beta_{\text{ER}}$ of *Culex* mosquitoes under different temperature settings were very limited [26,39,69]. The data were confined to the temperature range 15–30°C and while the data on *Cx. pipiens molestus* showed the most notable reduction in $\beta_{\text{ER}}$ at high temperature, the remaining data indicated that the trait is somewhat stable over the observed temperature range, although with tendencies for the highest trait values at intermediate temperatures (see Fig A in S1 Text). Overall, we found the information on $\beta_{ER}$ too scarce to establish and fit a suitable temperature-dependent function. Therefore, we decided to set this trait to a constant value determined by the mean across all observations in the dataset (given by 140 eggs per egg raft).

Our choice of functional forms for pathogen-related traits was primarily motivated by inspection of the available data (see section S1.1 in S1 Text for details). As a result, we modelled the mosquito infection probability $b_{M}\left(T\right)$ using a sigmoidal function:


$$\begin{array}{c} b_{M}\left(T;\alpha ,\beta \right)=\frac{1}{1+e^{-\left(\beta T+\alpha \right)}} \end{array}$$ (10)

As a model for the extrinsic incubation period $\text{EIP}\left(T\right)$, we chose an exponential decay function..


$$\begin{array}{c} EIP\left(T;\alpha ,\beta \right)=exp\left(-\beta T+\alpha \right) \end{array}$$ (11)

### Statistical inference of temperature-response functions

To estimate the temperature response function parameters for each trait, we implemented Bayesian hierarchical models. For mosquito-life history traits, our models include species and experiment identity as hierarchical levels of parameter estimates while we estimated the pathogen-related traits using experiment identity as the only hierarchical level. We assigned different experiment identities if the data came from separate articles or when an article investigated the temperature response of different populations of the same species (such as different strains [68], laboratory versus field-derived populations [44], or field-derived populations of different geographical origin [46]). Below, we provide a general summary of our models. The formal mathematical descriptions of our Bayesian models as well as prior and hyperprior specifications can be found in section SI3 in S1 Text.

#### Mosquito life-history traits.

For life-history traits, we modelled the trait performance $y_{ij,T}$ of species *i* in experiment *j* at a particular temperature *T* using a normal distribution likelihood with mean given by the trait-specific parametric function evaluated at the given temperature (see Equations (7)-(9)) with a parameter vector $\theta_{ij}$ (e.g., in case of juvenile development rate: $\theta_{ij}=\left(q_{ij},T_{\min\;ij},T_{\max\;ij}\right)$) depending on species and experiment identity and an estimated standard deviation *s*:


$$\begin{array}{c} y_{ij,T}|\theta_{ij},s\sim N\left(f^{X}\left(T;\theta_{ij}\right),s^{2}\right),X\in \left\{B,Q,L\right\} \end{array}$$ (12)

We assigned the model parameter vector $\theta_{ij}$ a multivariate normal hierarchical prior with mean parameter vector $\theta_{i}$, which represents the mean parameter realizations for species *i*, and diagonal covariance matrix defined by between-experiment standard deviations $\sigma^{\text{exp}}$, measuring the variability of parameter realizations across experiments on the same species:


$$\theta_{ij}\text{|}\theta_{i},\sigma^{\exp}\sim N\left(\theta_{i},\text{diag}\left(\sigma^{\exp2}\right)\right)$$ (13)

The mean parameter realizations $\theta_{i}$ are assigned another multivariate normal hierarchical prior


$$\begin{array}{c} \theta_{i}|\mu ,\sigma \sim N\left(\mu ,\text{diag}\left(\sigma^{2}\right)\right) \end{array}$$ (14)

with population-level means *μ*, reflecting the mean parameter realization across species, and between-species standard deviations *σ*, measuring the variability of parameter realizations across species.

We used the mean parameter realizations $\theta_{i}$ to calculate the expected trait temperature response of each species. We included the between-experiment variability $\sigma^{\text{exp}}$ since inspection of the data indicated a substantial variation in the temperature response between separate experiments on the same trait and species. Neglecting these experiment effects would ignore statistical dependencies within the data, which can lead to biased and overconfident model fits (see section SI5 in S1 Text).

Note that $\mu ,\sigma ,\sigma^{\text{exp}}$ are vectors whose dimensionality depends on the considered trait (e.g., in case of juvenile development rate: $\mu =\left(\mu_{q},\mu_{T_{\text{min}}},\mu_{T_{\text{max}}}\right)$). Our choice of diagonal covariance matrices implies that we assigned independent normal priors to the individual parameters in $\theta_{ij}$. To ensure that the estimates of the parameter *q* in case of the Briére and quadratic function as well as the parameter *β* in case of the linear model for mosquito adult lifespan are non-negative, we placed the normal hierarchical priors on log-transformed versions of these parameters. For simplicity of presentation, we neglected this detail in our notation above (see section SI3 in S1 Text for all details).

We defined the hyperpriors of the hierarchical prior parameters (population-level means, between-species standard deviations, and between-experiment standard deviations) dependent on trait data availability. We used vague hyperpriors in case of traits with extensive data (juvenile development rate and survival and partly adult lifespan). To regularize model fits for the less studied traits, we used informative priors that integrated prior knowledge on between-species and between-experiment standard deviations from biologically similar traits (see section SI3 in S1 Text for details).

#### Mosquito infection probability and extrinsic incubation period.

For the pathogen-related traits, we used a simpler hierarchical model with experiment identity as the only hierarchical level. We did not have sufficient data available to estimate parameters for groups of experiments on the same mosquito species or on the same mosquito species and virus strain combination. Data on the mosquito infection probability was available in binomial form. Therefore, we modelled the number $n_{j,T}$ of mosquitoes with a midgut infection (virus detected in body) upon the number $N_{j,T}$ of all mosquitoes tested in experiment *j* at temperature *T* using a binomial likelihood with success probability given by the sigmoidal function that we chose for this trait with parameter vector $\theta_{j}=\left(\alpha_{j},\beta_{j}\right)$:


$$\begin{array}{c} n_{j,T}|\theta_{j}\sim Bin\left(N_{j,T},b_{M}\left(T;\theta_{j}\right)\right) \end{array}$$ (15)

For the extrinsic incubation period, data were available as the percentage of “transmitting mosquitoes” (virus detected in salivary glands) upon all mosquitoes with a midgut infection (virus detected in body) dependent on the days post exposure *t* and the temperature setting *T*. We modelled the relationship between days post exposure *t* and the percentage $p_{j,t,T}$ of transmitting mosquitoes in experiment *j* at temperature *T* using a normal likelihood with an estimated standard deviation *s* and mean given by a cumulative Gaussian distribution Φ. We estimated the standard deviation $s_{\text{EIP}}$ of the cumulative Gaussian and modelled its mean (i.e., the point in time at which 50% of mosquitoes are expected to have a salivary gland infection) with the exponential decay function that we selected for the extrinsic incubation period trait:


$$\begin{array}{c} p_{j,t,T}|\theta_{j},s_{\text{EIP}},s\sim N\left(\text{Φ}\left(\text{t};\text{EIP}\left(T;\theta_{j}\right),s_{\text{EIP}}^{2}\right),s^{2}\right) \end{array}$$ (16)

For both pathogen-related traits, we assigned a multivariate normal hierarchical prior with population-level means *μ* and diagonal covariance matrix defined by between-experiment standard deviations $\sigma^{\text{exp}}$ to the parameter vector $\theta_{j}$:


$$\theta_{ij}\text{|}\mu \sim N\left(\mu ,\text{diag}\left(\sigma^{\exp2}\right)\right)$$ (17)

The population-level means of each parameter were assigned vague hyperpriors. However, due to limited data and lack of prior knowledge, it was impossible to derive reasonable estimates of the between-experiment standard deviations. Therefore, we fixed these parameters to specific values. These were chosen following a conservative approach that enforced some amount of pooling of the parameter estimates across experiments but also allowed for uncertainty of the temperature response estimate at the population-level (i.e., the temperature response generated by sampling from the hierarchical prior). Since we set these between-experiment standard deviations to subjectively chosen values, we investigated the sensitivity of our estimates to changes in these values (see SI4).

### Implementation and $R_{0}^{\text{rel}}$ calculations

We fitted the Bayesian models using Stan [87] through the *Rstan* package [88]. Stan provides full Bayesian inference via Markov chain Monte Carlo (MCMC) methods. Here, we used the No-U-Turn sampler, a form of Hamiltonian Monte Carlo sampling. For each trait, we ran 4 MCMC chains for 4000 iterations, of which 2000 iterations served as warmup. We checked the Markov chains for convergence using the Gelman-Rubin statistic and by visual inspection (see section SI10 in S1 Text). For the six *Culex* species, we obtained species-specific posterior estimates for $R_{0}^{\text{rel}}$ by applying the posterior samples of the expected temperature response of each trait to Equation (2). From this posterior distribution, we calculated mean estimates and 95% credible intervals (CIs) for the temperature limits $T_{\text{min}}^{R}$, $T_{\text{max}}^{R}$ and the temperature optima $T_{\text{opt}}^{R}$ of WNV transmission suitability for each species.

To build the $R_{0}^{\text{rel}}\left(T\right)$ model for species that lacked data on one or more life-history traits, we substituted missing temperature response estimates by population-level estimates generated by sampling parameter values from the hierarchical prior $N\left(\mu ,\text{diag}\left(\sigma^{2}\right)\right)$ (Equation (14)). In the context of our Bayesian models, these hierarchical priors represent the uncertainty about a species’ expected trait temperature response in the absence of data on this specific species (see Fig B in S1 Text). For mosquito infection probability, we used the population-level estimates generated by sampling from the hierarchical prior $N\left(\mu ,\text{diag}\left(\sigma^{\exp2}\right)\right)$ (Equation (17)) in each species’ $R_{0}^{\text{rel}}$ model since for this trait only data for *Cx. pipiens* was available. In case of the extrinsic incubation period, we used the fits derived for WN02 in *Cx. pipiens* in the $R_{0}^{\text{rel}}\left(T\right)$ model for *Cx. pipiens*, NY99 in *Cx. tarsalis* for *Cx. tarsalis*, and the population-level estimates for all other species (see Fig C in S1 Text).

To understand the contribution of each mosquito-pathogen trait to the $R_{0}^{\text{rel}}\left(T\right)$ estimates, we implemented a sensitivity and uncertainty analysis. First, we recalculated $R_{0}^{\text{rel}}\left(T\right)$ for each species while keeping single mosquito-pathogen traits constant to see which traits were important for determining the temperature limits and temperature optima of $R_{0}^{\text{rel}}\left(T\right)$. Then, we investigated the contribution of each trait to the uncertainty of $R_{0}^{\text{rel}}\left(T\right)$ by means of a Sobol’ analysis. To this end, we calculated first-order and total-effect Sobol’ indices [89,90] for each trait for temperatures between 15°C and 30°C using the *sensitivity* package in R [91]. Details on this analysis are provided in section SI11 in S1 Text.

## Results

Here, we present the results on temperature suitability and estimates for the expected trait temperature response for the main species of interest in the transmission of WNV: *Cx. pipiens, Cx. quinquefasciatus, Cx. pipiens molestus, Cx. pipiens pallens, Cx. restuans, Cx. tarsalis*. An overview of all trait temperature response parameter estimates including other species can be found in Tables A-H in S1 Text.

### $R_{0}^{\text{rel}}$ transmission suitability temperature response

**Fig 3:**
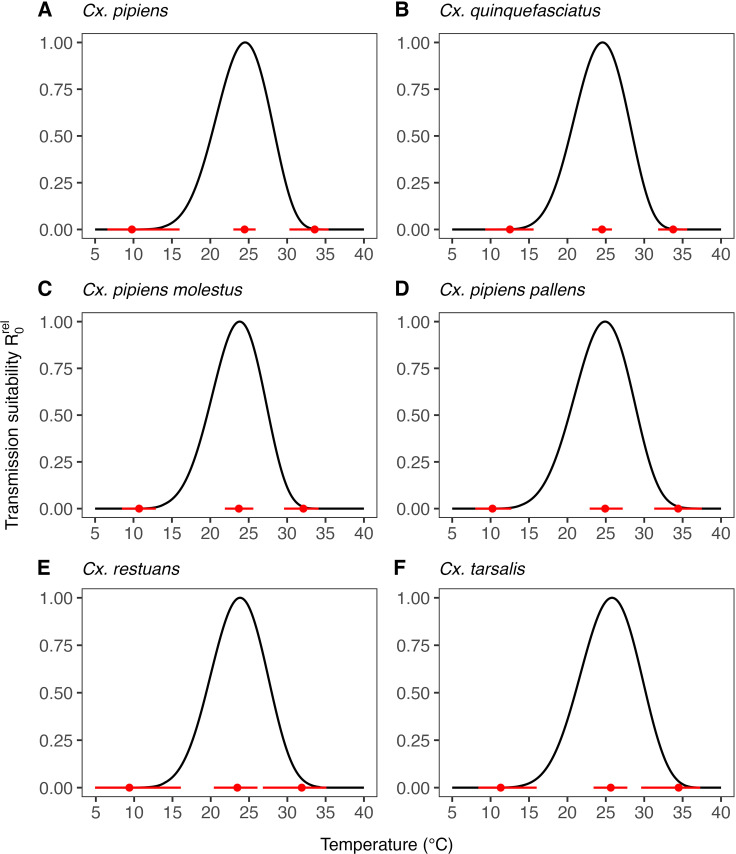
Estimated temperature response of the relative basic reproduction number $R_{0}^{\text{rel}}$ for WNV in six *Culex* species. Black solid lines indicate the mean temperature response. Red dots indicate mean estimates of the optimal temperature and temperature limits of $R_{0}^{\text{rel}}$. Red solid lines represent the corresponding central/equal-tailed 95% CI.

Across the six *Culex* species considered here, we find similarities in the temperature-sensitivity of $R_{0}^{\text{rel}}$ (Fig 3 and Table 2). We predict that WNV transmission suitability peaks at moderate temperatures with mean estimates across species ranging from 23.5°C to 25.6°C and broadly overlapping 95% CIs. Our estimates of temperature limits are similarly consistent between species with the lowest estimate for the upper temperature limit at 31.9°C (95% CI: 26.8–35.1°C) for *Cx. restuans* and the highest at 34.5°C for *Cx. tarsalis* (95% CI: 29.6–37.3°C). The estimates for lower temperature limits range from the lowest estimate for *Cx. restuans* at 9.4°C (95% CI: 4.9–16.1°C) to the highest estimate for *Cx. quinquefasciatus at* 12.5°C (95% CI: 9.3–15.6°C)*.* Despite the observed similarity in these estimates, the associated uncertainties leave room for considerable differences in the temperature response across species. Overall, the estimated uncertainty in temperature limits is larger than in temperature optima for transmission. Because some species lacked data on a specific trait, we substituted their temperature response with a population-level estimate generated by sampling from hierarchical priors. Consequently, the uncertainty in temperature optima and limits varies by species depending on trait data availability. Among all species, *Cx. restuans* exhibits the highest uncertainty as it lacked specific data on adult biting rate, egg viability, and pathogen-related traits, and had limited data available on adult mosquito lifespan.

**Table 2:** Overview of our estimates of the optimal temperature and temperature limits of $R_{0}^{\text{rel}}\left(T\right)$ for WNV in *Culex* species.

Species	$T_{min}^{R}$	$T_{opt}^{R}$	$T_{max}^{R}$
*Cx. pipiens*	9.8 (6.6–16)	24.5 (23.0–25.9)	33.6 (30.3–35.4)
*Cx. quinquefasciatus*	12.5 (9.3–15.6)	24.5 (23.2–25.8)	33.8 (31.8–35.6)
*Cx. pipiens molestus*	10.7 (8.5–12.9)	23.7 (21.9–25.6)	32.1 (29.6–34.1)
*Cx. pipiens pallens*	10.2 (8.0–12.7)	24.9 (22.9–27.2)	34.4 (31.3–37.5)
*Cx. restuans*	9.4 (4.9–16.1)	23.5 (20.4–26.1)	31.9 (26.8–35.1)
*Cx. tarsalis*	11.3 (8.4–16.0)	25.6 (23.4–27.8)	34.5 (29.6–37.3)

The results of our sensitivity and uncertainty analysis (see section SI11 in S1 Text) indicate that the mosquito-pathogen traits differ in importance for determining the temperature limits and optima of transmission suitability $R_{0}^{\text{rel}}$. Keeping single traits constant and recalculating $R_{0}^{\text{rel}}$ (see Figs K-P in S1 Text) shows that the temperature response estimates of egg viability (for all species but *Cx. pipiens molestus* and *Cx. pipiens pallens*) and juvenile survival are most influential on the lower temperature limit estimates of $R_{0}^{\text{rel}}$. The upper temperature limits of $R_{0}^{\text{rel}}$ are primarily driven by including the temperature response estimates of adult mosquito lifespan (for all species but *Cx. tarsalis*), egg viability (for *Cx. pipiens*, *Cx. pipiens molestus, Cx. tarsalis*), and juvenile survival (for *Cx. restuans* and *Cx. tarsalis*). The temperature response estimates of adult mosquito lifespan, biting rate, and juvenile development rate are the most influential on the temperature optimum of $R_{0}^{\text{rel}}$. The Sobol’ analysis (see Figs Q-U in S1 Text) reveals the contribution of each trait to the uncertainty in $R_{0}^{\text{rel}}$, which varies by species and temperature. Overall, biting rate is the primary source of uncertainty for all species except for *Cx. pipiens pallens*, for which adult mosquito lifespan has a greater impact. At high temperatures, adult mosquito lifespan becomes the dominant contributor to uncertainty in $R_{0}^{\text{rel}}$ except for *Cx. tarsalis*, where biting rate remains the most important. All other traits contribute less with further variations across species and temperature ranges. For instance, egg viability often plays a larger role at extreme temperatures, whereas juvenile development rate tends to contribute more at intermediate temperatures, except for *Cx. pipiens*, where egg viability remains more influential and *Cx. pipiens pallens*, where juvenile development rate is consistently more important. Across all species and temperatures, juvenile survival consistently contributes the least to uncertainty in $R_{0}^{\text{rel}}$.

### Impact of mechanistic model assumptions on transmission suitability estimates

**Fig 4:**
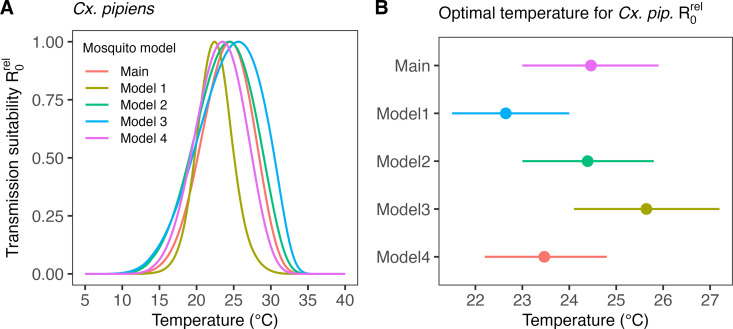
(A) Mean temperature response of the relative basic reproduction number $R_{0}^{\text{rel}}$ for WNV in *Cx. pipiens* contrasting the estimate from transmission suitability model introduced in the main text (see Equation (5)) as well as from four alternative model formulations from the published literature (see Equations (SI7.1–7.7) in S1 Text). (B) Mean estimates of the optimal temperature of $R_{0}^{\text{rel}}$ per model (dots) and the corresponding central/equal-tailed 95% CIs (lines).

We tested whether changes to mechanistic assumptions underlying the $R_{0}^{\text{rel}}\left(T\right)$ model would impact our results on WNV temperature suitability. Our main model incorporates the impact of temperature on mosquito abundance suitability through an equilibrium expression derived from a mosquito population dynamics model. We compared the results for *Cx. pipiens* using this model against four alternative models. Three alternative models use different mosquito equilibrium expressions derived from mosquito population dynamic models, each varying in their representation of mosquito intra-specific competition. The fourth alternative model uses a mosquito abundance expression previously used in various studies on MBD thermal biology [19,20,22,23,25,28–30,32], which lacks a formal connection to a mosquito population dynamics model (see section SI7 in S1 Text). Fig 4 demonstrates a notable impact of mosquito modelling assumptions on the transmission suitability temperature response curve. Across the different models, the mean estimates for the temperature optimum for WNV suitability transmitted by *Cx. pipiens* vary by up to 3.0°C, with the estimate from our main model falling between those of the alternative models. The estimates of the WNV suitability temperature optimum by model are given by: 24.5°C (95% CI: 23.0–25.9°C) for the main model (using Equation (5)), 22.6°C (95% CI: 21.5–24.0°C) for alternative model 1 (using Equation (SI7.2) in S1 Text), 24.4°C (95% CI: 23.0–25.8°C) alternative model 2 (using Equation (SI7.4) in S1 Text), 25.6°C (95% CI: 24.1–27.2°C) alternative model 3 (using Equation (SI7.6) in S1 Text), and 23.5°C (95% CI: 22.2–24.8°C) for alternative model 4 (using Equation (SI7.7) in S1 Text).

### Temperature response of mosquito life-history traits

**Fig 5:**
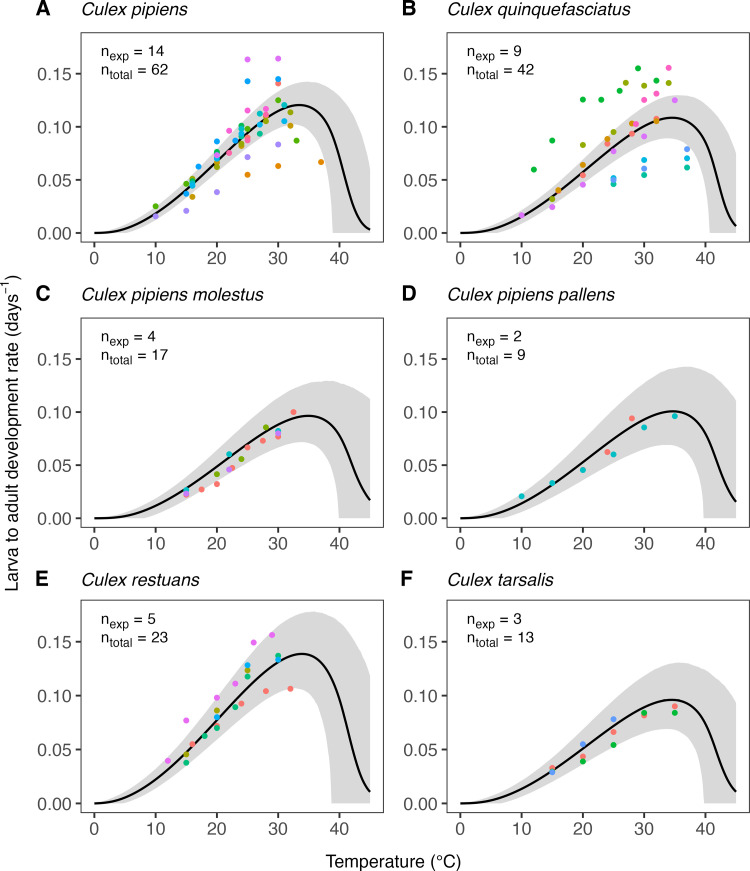
Estimates of the expected temperature response of the larva to adult development rate for six *Culex* species. Dots represent data from experimental studies, with measurements for the same species shown in different colours to indicate separate experiments. Black solid lines represent posterior distribution mean model fits. Grey shaded areas represent the corresponding central/equal-tailed 95% CI. n_exp_ and n_total_ denote the number of experiments and the total number of data points for each species.

**Fig 6:**
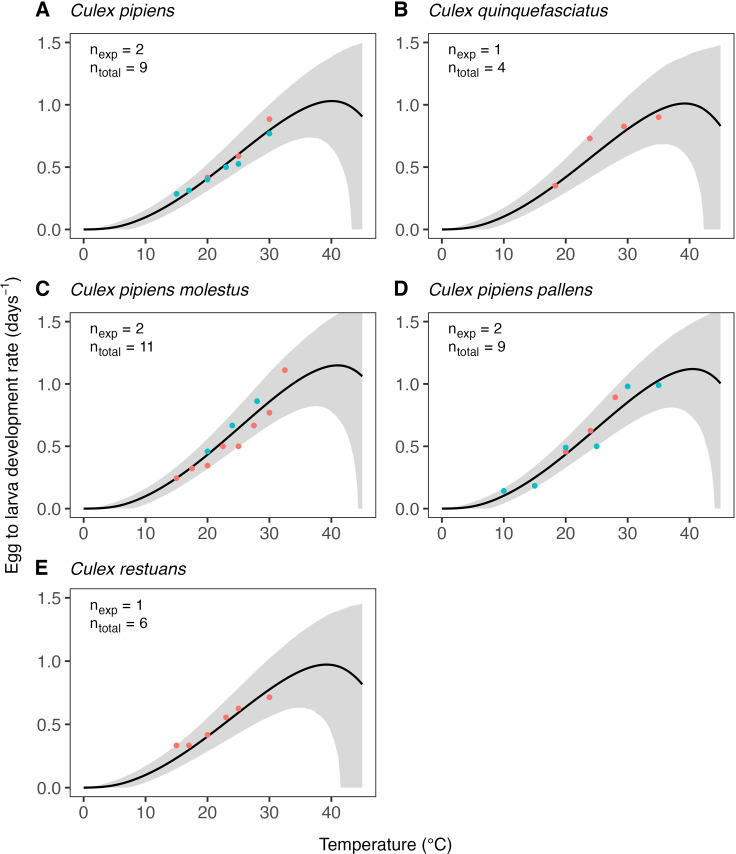
Estimates of the expected temperature response of the egg to larva development rate for five Culex species. Dots represent data from experimental studies, with measurements for the same species shown in different colours to indicate separate experiments. Black solid lines represent posterior distribution mean model fits. Grey shaded areas represent the corresponding central/equal-tailed 95% CI. nexp and ntotal denote the number of experiments and the total number of data points for each species.

**Fig 7:**
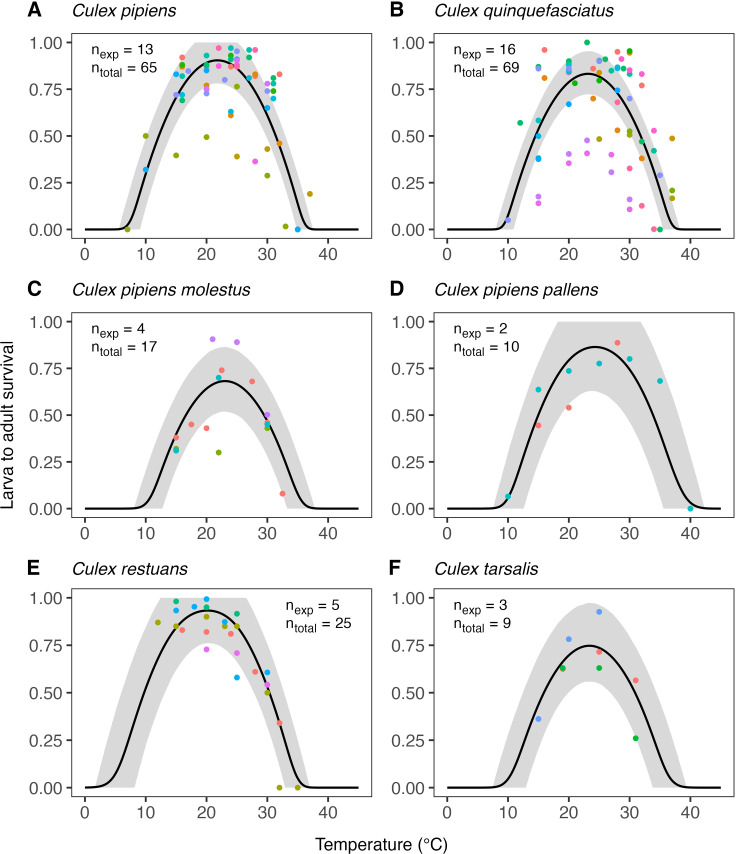
Estimates of the expected temperature response of larva to adult survival for six *Culex* species. Dots represent data from experimental studies, with measurements for the same species shown in different colours to indicate separate experiments. Black solid lines represent posterior distribution mean model fits. Grey shaded areas represent the corresponding central/equal-tailed 95% CI. n_exp_ and n_total_ denote the number of experiments and the total number of data points for each species.

**Fig 8:**
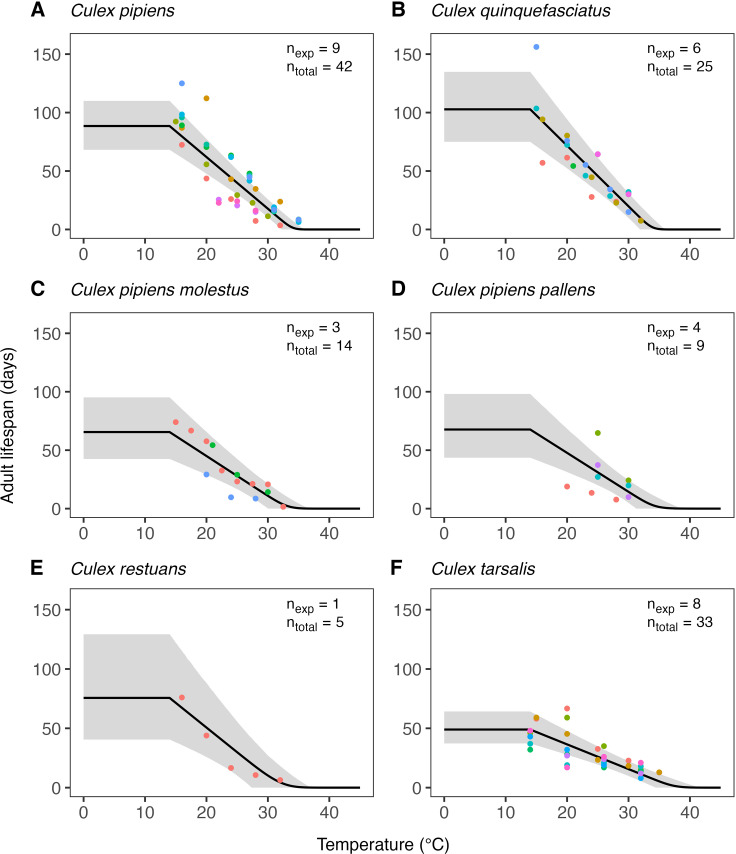
Estimates of the expected response of adult mosquito lifespan for six *Culex* species. Dots represent data from experimental studies, with measurements for the same species shown in different colours to indicate separate experiments. Black solid lines represent posterior distribution mean model fits. Grey shaded areas represent the corresponding central/equal-tailed 95% CI. n_exp_ and n_total_ denote the number of experiments and the total number of data points for each species.

**Fig 9:**
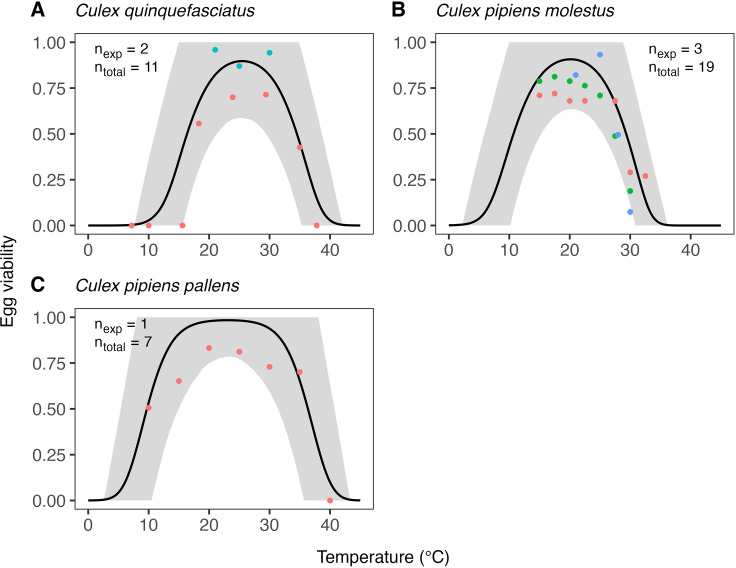
Estimates of the expected temperature response of egg viability for three Culex species. Dots represent data from experimental studies, with measurements for the same species shown in different colours to indicate separate experiments. Black solid lines represent posterior distribution mean model fits. Grey shaded areas represent the corresponding central/equal-tailed 95% CI. nexp and ntotal denote the number of experiments and the total number of data points for each species.

**Fig 10:**
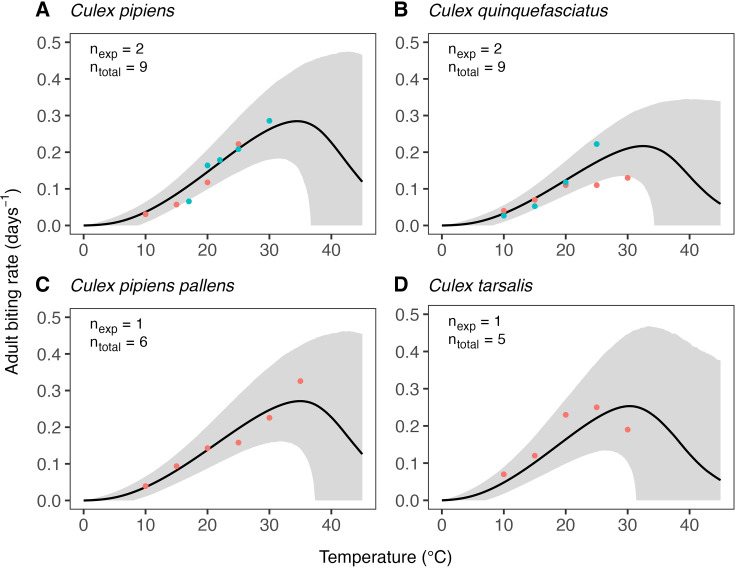
Estimates of the expected temperature response of the adult biting rate for four Culex species. Dots represent data from experimental studies, with measurements for the same species shown in different colours to indicate separate experiments. Black solid lines represent posterior distribution mean model fits. Grey shaded areas represent the corresponding central/equal-tailed 95% CI. nexp and ntotal denote the number of experiments and the total number of data points for each species.

The estimates of the expected temperature responses for each life-history trait are shown in Figs 5–10. An overview of the corresponding posterior mean parameter estimates and 95% CIs for each life-history trait can be found in Tables A-F in S1 Text. The model fits for juvenile development rates (Fig 5), and similar for egg development rates (Fig 6), indicate relatively high temperature optima with mean estimates for juveniles ranging 33.7-35.4°C across the six *Culex* species. Furthermore, temperature limits estimates are outside the limits dictated by other traits with upper limit estimates ranging 41.9–43.8°C and lower limits 0.6–3.0°C. Due to very limited data available on the development rates at high temperatures, we estimate substantial uncertainty in the temperature response of these traits in the high-temperature regimes. In contrast to juvenile development rates, the model fits for juvenile survival (Fig 7) indicate lower temperature optima (mean estimates ranging 20.0–24.4°C) and tighter temperature limits across the six species (upper limits ranging 34.3–38.8°C and lower 5.1–10.6°C). The temperature response fits for adult lifespan (Fig 8) indicate that some species, e.g., *Cx. pipiens* and *Cx. quinquefasciatus*, have substantially higher lifespan at lower temperatures than others, e.g., *Cx. tarsalis*. The estimate of the upper temperature limit was highest for *Cx. tarsalis* with 37.7°C (95% CI: 34.4–41.5) and lowest for *Cx. restuans* with 32.5 (95% CI: 27.3–37.0). No data were available for this trait below 14°C. As a conservative approach to modelling trait performance, we plateaued the adult lifespan fits below this threshold. This method limits the impact of adult lifespan on $R_{0}^{\text{rel}}\left(T\right)$ in temperature regimes where it was not measured but also implies that the estimates of this trait below 14°C should be interpreted with caution. For egg viability (Fig 9), data from only three of the *Culex* species were available (and of four species in total), which resulted in a relatively large uncertainty in model fits. Our estimates for the temperature limit parameters of this trait range 33.3–39.5°C for the upper limits and 6.6–12.3°C for the lower limits across the three species. Temperature optima range from 20.0–25.4°C. The estimates of the temperature sensitivity of biting rates (Fig 10) were also limited by the available data and therefore resulted in a relatively large uncertainty. Our fits indicate that the upper temperature limit parameter for this trait range between 40.8–45.7°C, the lower temperature limit 0.2–1.9°C, and the temperature optimum 32.7–36.7°C between species. Similar as for development rates, data on adult biting rate were scarce at high temperatures, resulting in particularly high uncertainty in model fits for these settings. The 95% CIs of parameter estimates largely overlap across species for all traits. The population-level temperature responses that we generated by sampling parameters from the hierachical priors determined by the estimated population-level means and between-species standard deviations show a relatively large uncertainty in our expectation of the trait temperature responses of a new species in the absence of data (Fig B in S1 Text).

For the juvenile development rate, the estimate of the between-experiment standard deviation for the temperature limit parameters of the Briére function is larger than the between-species standard deviation. Conversely, the between-species standard deviation is larger for the parameter *q* of the same function. In contrast, for juvenile survival, we estimate a higher variability in the temperature limit parameters of the quadratic function between species than between experiments, and the opposite is true for the parameter *q*. For the slope parameter of the linear function for mosquito adult lifespan, we estimate a greater variability across species than between experiments. Despite these differences, the 95% CIs for between-species and between-experiment standard deviations largely overlap for all three traits. The estimates of all other between-species and between-experiment variabilities show similar patterns. However, due to data scarcity, these estimates were derived using informative hyperpriors to regularize parameter estimation.

### Temperature response of pathogen-related traits

**Fig 11:**
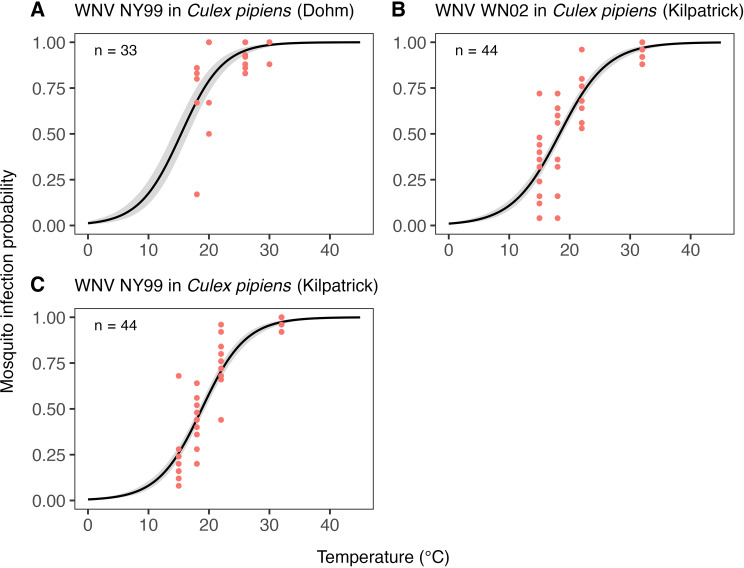
Estimates of the expected temperature response of the mosquito infection probability for three experiments testing different WNV strains in Cx. pipiens. Dots represent data from experimental studies. Black solid lines represent posterior distribution mean model fits. Grey shaded areas represent the corresponding central/equal-tailed 95% CI. n denotes the number of data points for each experiment.

**Fig 12:**
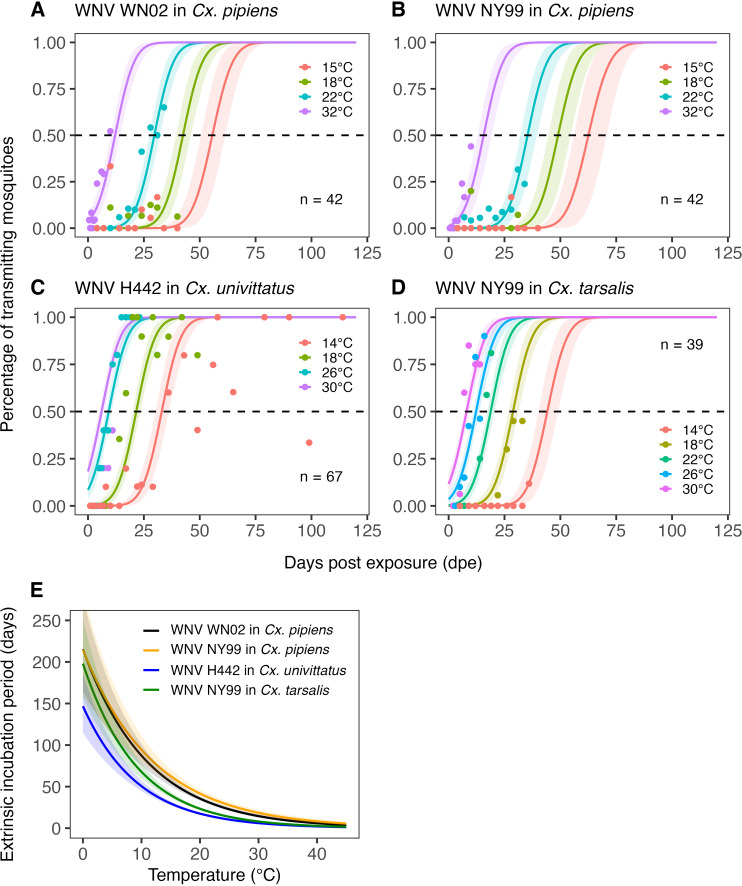
(A-D) Estimated relationship between the days post exposure and the percentage of transmitting mosquitoes upon infected mosquitoes for four different experiments each testing multiple temperatures. (E) Corresponding estimates of the expected temperature response of the extrinsic incubation period (in our statistical model defined as the time when 50% transmitting mosquitoes are expected to be reached, see Equation (16)) for the four different experiments. Solid lines represent posterior distribution mean model fits. Shaded areas represent the corresponding central/equal-tailed 95% CI. n denotes the number of data points for each experiment.

Figs 11 and 12 show the estimated temperature responses of the mosquito infection probability and extrinsic incubation period, respectively. Posterior mean parameter estimates and 95% CIs for the pathogen-related traits can be found in Tables G-H in S1 Text. For mosquito infection probability, only data for two WNV strains in *Cx. pipiens* was available. The sigmoidal model describes well the monotonic increase of this trait with temperature observed in the data. For the extrinsic incubation period, data were available for three different WNV strains tested in *Cx. pipiens, Cx. univittatus,* or *Cx. tarsalis.* The experiment-level estimates shown in Fig 12E indicate differences in the extrinsic incubation period across the different experiments. The experiment on WNV H442 in *Cx. univittatus* is estimated to have the shortest extrinsic incubation period across all temperature settings, followed by WNV NY99 in *Cx. tarsalis,* WNV WN02 in *Cx. pipiens,* and WNV NY99 in *Cx. pipiens*. Our exponentially decreasing model for the extrinsic incubation period is generally well in line with the available data. Only in the case of WNV H442 in *Cx. univittatus* do the data suggest an increase of the extrinsic incubation period at high temperatures, which resulted in a poor model fit for the 30°C setting for this experiment (Fig 12C). Due to the limited data available, we fixed the between-experiment standard deviation of parameters for these traits and could not estimate it. As a result, the population-level temperature responses generated for these traits (see Fig C in S1 Text) should be seen as provisional. We investigated the sensitivity of our results to this data gap in section SI4 in S1 Text.

## Discussion

Understanding how mosquito-borne disease transmission responds to changes in climatic conditions is critical for climate change adaptation and mitigation. This study provides novel estimates for the temperature response of WNV transmission suitability using a relative version of the basic reproduction number $R_{0}^{\text{rel}}\left(T\right)$. We constructed Bayesian hierarchical models to estimate species-specific temperature response functions from experimental data for several mosquito-pathogen traits influencing transmission suitability. This new framework facilitates partial pooling of data to regularize temperature response fits of species with sparse data and avoids biases introduced when pooling data across experiments on the same species. For this novel multi-species analysis, we updated a dataset compiling outcomes of laboratory studies that test trait responses to temperature. While the dataset included various other mosquito species that helped to estimate hierarchical priors and regularize model fits, our analysis focused on *Culex* mosquitoes and their potential to transmit WNV.

### Results in context

Our results suggest that WNV transmission suitability is optimized around 24°C with similar estimates across all species considered. These results are in line with some previous estimates of WNV temperature suitability [15,20,24–26] but notably lower than others [27,92] (see Table K in S1 Text). We estimate the highest lower temperature limit for *Cx. quinquefasciatus*, which could be explained by the more tropical distribution range of this species [3]. However, we estimate two other species, *Cx. pipiens pallens* and *Cx. tarsalis*, to have higher temperature optima and upper temperature limits. In addition, the 95% CIs of temperature limits and the temperature optimum of all species are largely overlapping. The population-level temperature response estimates substituted when species-specific trait data was lacking led to relatively large uncertainties in the transmission suitability estimates for some species. The estimated uncertainty is generally higher in temperature limits than temperature optima for transmission. Overall, our analysis suggests similarities in the temperature response for all six considered *Culex* species, but considering the uncertainties in some of our estimates, the data currently do not allow to draw definitive conclusions about the degree of similarity. Considerable differences between species remain a possibility, particularly in temperature limits. Such differences, even if small, could have significant impacts under climate change.

A direct comparison between temperature limits estimated in our study and previous studies on WNV temperature suitability is difficult due to conceptual differences in the modelling approaches and in trait definitions. Our $R_{0}^{\text{rel}}\left(T\right)$ model is zero if and only if the mosquito population equilibrium $M^{*}\left(T\right)$ is zero, determined by the mosquito life-history traits. This contrasts with previous studies [15,20,25,26] where pathogen-related traits were not modelled as strictly positive and could therefore affect the temperature limits of $R_{0}^{\text{rel}}\left(T\right)$. Therefore, the temperature limits identified in our study have a clear interpretation as limits for the stability of active mosquito populations. To note, these limits only apply if temperatures stay outside them for an extended period. Moreover, the *Culex* mosquitoes considered here use different forms of dormancy (such as diapause in *Cx. pipiens* and quiescence in *Cx. quinquefasciatus*) to persist periods of cold temperatures. While there is certainly no transmission risk during these periods, active mosquito populations can reemerge once temperature conditions become more favourable.

### Sensitivity of the optimal transmission temperature

We integrated the impact of temperature on mosquito abundance into the transmission suitability model using an equilibrium expression derived from a mosquito population dynamics model. Several previous studies on mosquito thermal biology have used a similar approach but based their mosquito abundance expression on an erroneous equilibrium derivation (see section SI7 in S1 Text) [19,20,22,23,25,26,28–30,32,61]. We compared our result on temperature suitability for WNV in *Cx. pipiens* to a model that uses this previous expression and to equilibria derived from alternative mosquito population dynamic models that differ in their representation of intra-specific competition [55–60]. We show that the essential estimate of the optimal temperature for transmission suitability can vary by 3°C across these models. While this variation does not question that WNV transmission suitability peaks at moderate temperatures, a 3°C difference is within the range of mean temperature changes by the end of century in suboptimal climate change scenarios and could therefore heavily impact WNV risk change projections. The primary model introduced in the main text draws on standard model formulations in mosquito ecology but is still a highly simplified representation of reality. For this reason, we cannot conclusively assert that our predictions for the optimum temperature are the most accurate. Currently, we emphasize the need to further investigate this sensitivity, to transparently outline the assumptions and mechanisms underlying modelling choices, and to scrutinize these models.

### Advantages of Bayesian hierarchical models

For the estimation and multi-species comparison of trait temperature responses, as considered in this study, the Bayesian hierarchical models introduced here have important advantages over the statistical models used in previous studies on the thermal biology of MBDs [20,22,23,25,28,29]. Hierarchical priors offer a purely data-driven approach to introduce partial pooling at the species-level (and experiment-level), effectively regularizing model fits in cases with sparse data. Moreover, our approach accounts for both between-species and between-experiment variability of temperature responses, acknowledging statistical dependencies within the dataset. In addition, the hierarchical priors are interpretable, and by sampling from them, we can generate uncertainty estimates for the temperature response of mosquito species that lack trait data.

Earlier studies [20,22,29] instead used a sequential approach to partial pooling. First, empirical prior distributions for a focal species were derived from the data pooled across the experiments on all other species. In some cases, this pooled dataset resulted in overly restrictive priors which were adjusted by variance inflation. Subsequently, the data of the focal species were pooled across experiments (see also [23,25,28]) and used alongside the constructed priors for posterior estimation. The process was repeated for each species. This approach has major limitations: (i) Assigning different prior distributions to each species in the dataset lacks logical consistency and prevents generalizability to new species that currently lack data. (ii) The approach is not fully Bayesian, as it involves point estimates for the parameters of the prior distributions. (iii) The choice of variance inflation is subjective, impairing reproducibility and potentially biasing posterior estimates. (iv) Pooling data across experiments disregards statistical dependencies related to experiment identity, which can bias temperature response estimates and uncertainty quantification (see section SI5 in S1 Text). The new approach introduced here solves these issues.

### Meaning and implications of between-experiment variability

For a few life-history traits (juvenile development rate, juvenile survival, and partly for mosquito adult lifespan), we had sufficient data to estimate the between-species and between-experiment variability of parameter estimates using vague hyperpriors. Our results indicate that trait temperature responses exhibit similar variability across species as across experiments on the same species. While we observe potential differences in the relative importance of these variabilities among traits, further data are needed to confirm these findings. Variability in the trait temperature response across experiments on the same species may, for instance, arise due to unaccounted differences in experimental setups (such as differences in food supply for juvenile development), intraspecific genetic variation, or phenotypic plasticity. Regardless of the reason, this variability suggests that outcomes from single experiments may not align with the expected temperature response of a species and ultimately hinder comparisons between species. Our statistical approach accounts for between-experiment variability to get unbiased estimates of the expected temperature responses of each species. However, we did not yet explore the causes of this variability and focused our analysis of transmission suitability on the expected trait temperature responses. Therefore, our estimates of $R_{0}^{\text{rel}}\left(T\right)$ do not reflect the between-experiment variability, suggesting that mosquito populations in different field settings may exhibit temperature responses that could lead to greater variation in transmission suitability. For a specific study location, our estimates can serve as a valuable prior assumption but could probably be refined by experimentation on the local mosquito populations and circulating WNV strains. The potential for intraspecific temperature response variation is underlined by a recent study [26] looking at two distinct *Cx. pipiens* populations from New York State, which showed variation in life history trait temperature responses between the populations consistent with local temperature conditions and WNV prevalence.

### The need for more experimental data

The data on other traits were too scarce to estimate the hierarchical model without including prior knowledge (egg viability, egg development rate, adult biting rate, and partly adult lifespan) or reducing model complexity (mosquito infection probability and extrinsic incubation period). Additional experimental data on these traits are thus needed to allow less restrictive modelling assumptions and derive more accurate estimates of how they vary across mosquito species, experiments, and WNV strains. While we would initially suggest focusing data collection to cover all relevant temperatures on a single species (and virus strain), data on other species (and virus strains) and across experiments are ultimately needed to investigate the generalizability of these trait estimates.

One priority should be on vector competence studies using mosquito populations and WNV strains of different origins to investigate potential differences to the ones currently represented in our data. These should optimally not only test multiple temperature settings but also sample across multiple different time points post exposure to allow estimation of sophisticated temperature response functions. For example, our dataset currently lacks such studies on European mosquitoes and WNV strains (see Table J in S1 Text), despite the importance of WNV in Europe. Several WNV vector competence studies have been conducted with European mosquitoes and WNV strains but to the best of our knowledge all of them either test fewer than three temperatures or sample only once or twice post exposure [93–99], limiting the use of their results for mathematical modelling.

To reduce the overall uncertainty in the transmission suitability estimates, our sensitivity and uncertainty analyses suggest that adult mosquito biting rate and lifespan should be the priorities for future experimental studies. As a result of the combination of the sensitivity of $R_{0}^{\text{rel}}$ to these traits and the uncertainty in their temperature response estimates, they contribute the most to uncertainty in the current $R_{0}^{\text{rel}}$ estimates. Therefore, they provide the greatest leverage for reducing uncertainty. However, our results do not indicate the amount of additional data required to achieve a reduction in uncertainty for one trait relative to others, which should generally be lower for highly uncertain traits. Therefore, it should be sensible to prioritise experimental studies on traits that currently have the least data available, thus, prioritizing biting rate and for some species even egg viability over adult lifespan. If the aim is to get more precise estimates of the temperature limits and optima of transmission suitability, our suggestions can be refined. For the lower temperature limits, our results suggest that measurements of egg viability and, to lesser extent, juvenile survival at low temperatures should be key priorities. For upper temperature limits, more precise estimates of adult mosquito lifespan at high temperatures are essential, along with egg viability and juvenile survival to a minor extent. To improve estimates of temperature optima, adult mosquito lifespan should be prioritized, followed by biting rate and juvenile development rate. The exact suggestions vary by species, and the result of our sensitivity and uncertainty analysis (see Figs K-U in S1 Text) can help guide targeted efforts.

Independently of transmission suitability, the estimates of some traits could be improved with new experimental data. For example, there is a lack of data on juvenile development rate, egg hatching rate, and adult biting rate at extreme high temperatures where these traits are hard to measure due to high mortality. Our Bayesian framework allows us to explicitly represent and quantify the increased uncertainty in these temperature regimes but the estimated response shape around and beyond the temperature optimum of these traits is still strongly influenced by the choice of functional form rather than direct data constraints. The functional form that we chose for these traits is based on biological theory but additional data in the high temperature regimes would allow to improve model accuracy. Similarly, there is a lack of data on mosquito adult lifespan at low temperatures where we simply plateaued the temperature response of this trait. While the estimates of these traits at these temperatures have no or limited impact on our static transmission suitability measure, they might become important in dynamic simulations. It is important to realize that a low sensitivity of $R_{0}^{\text{rel}}$ to some traits does not imply that these traits are unimportant for accurately describing disease dynamics, including delays in the mosquito life and WNV transmission cycles.

### Benefits from standardized data reporting

Data across the experimental studies gathered in our database were reported quite heterogeneously. While some data were available as numbers in tables or text, we had to extract a significant amount of data from figures using a plot digitizer tool [100]. Moreover, these data either represented summary statistics across outcomes of multiple replicates (e.g., [46,68,77]) or were the outcome of a single replicate (e.g., [36,80]). The number of replicates, the sample size within each replicate, and measures of uncertainty were often not fully reported. These data limitations restricted our modelling possibilities. For instance, we approximated juvenile survival and egg viability using a normal distribution likelihood. A binomial distribution likelihood would be a more suitable model for these traits but would have required access to raw data (sample size and number of successes) as we had available for the mosquito infection probability. Overall, more standardized data reporting across mosquito temperature response experiments would be highly valuable for the subsequent use of this data for modelling and the synthesis of results across studies. Preferably, experimental studies would avoid reporting composite measures of mosquito-pathogen processes and report the full empirical distribution for each process [85].

### Constant versus fluctuating temperatures

Our analysis has several limitations. We used the relative basic reproduction number as a static measure of transmission suitability under constant temperatures. This model captures the nonlinear effects of constant temperatures on the different mosquito-pathogen traits and can be used to detect optimal temperature conditions for disease transmission assuming a static environment. Similar approaches have been widely adopted to different mosquito-pathogen systems and successfully applied to predict general patterns of disease occurrence [19,20,22,25,26]. Nonetheless, this approach might fail to capture additional nonlinear effects created by temperature fluctuations which naturally occur in space and time. This raises the question of how applicable our results and models are to real-world scenarios, both to predict individual mosquito-pathogen trait performance and transmission suitability. This question is subject to current research in insect thermal biology and has not yet been conclusively clarified [19,31,101]. As a result of Jensen’s inequality and of the nonlinear temperature response of mosquito-pathogen traits and transmission suitability, estimates for the same mean temperature are expected to alter for different forms of temperature fluctuations around the mean [31,102,103]. At the trait-level, this difference can directly be predicted from our temperature response estimates by rate summation applied to the instantaneous rates that are directly (e.g., development rates) or indirectly (e.g., mortality rates derived from survival probabilities) connected to the mosquito-pathogen traits. If rate summation fails to predict trait performance under fluctuating conditions, fluctuating temperatures would have additional influences on trait performance through mechanisms such as stress accumulation, temperature acclimation, repair mechanisms during exposures to favourable temperatures, or ontogenetic shifts [101]. There is evidence that insect development rates, for example, can be accurately predicted via rate summation from experimental data generated under constant temperature conditions [101], while the same might not be true for survival, where constant temperature exposures might fail to capture time-dependent, non-lethal effects from which individuals might recover when returned to more favourable conditions [19,101,104,105]. A comprehensive analysis under which conditions rate summation can accurately predict mosquito-pathogen trait performance is currently lacking [20]. We want to note that using trait performance data from laboratory studies conducted under fluctuating instead of constant temperatures to fit temperature response functions [28] makes rate summation and therefore prediction of other fluctuation scenarios inapplicable. To relax assumptions of constant temperature in the transmission suitability model, analytical approaches developed under assumptions of periodic environments could be applied in future studies [106,107]. Furthermore, our mosquito-pathogen trait response functions could also be used to drive simulations of dynamic models. This would allow to investigate the impact of any form of temperature fluctuations on mosquito population and disease dynamics given fine-resolution temperature input data, relying on the assumption that rate summation offers accurate predictions. In contrast to these more complex approaches the $R_{0}^{\text{rel}}$ model probably has less explanatory power but represents a general model to compare temperature suitability across mosquito-pathogen systems [19].

### Laboratory versus field trait performance

Laboratory estimates of mosquito-pathogen traits can differ significantly from field performance. For example, it is well known that adult mosquito lifespan is substantially shorter in the field than under laboratory conditions [108,109]. Similarly, the use of lab-colonized mosquito populations, partly applying to our dataset (see Table J in S1 Text), can impact trait performance. This is a well-known issue for vector competence estimates [95,110,111] where field estimates often show a complicated picture of geographic and temporal variation [111,112].

### The multi-driver context of WNV transmission

Our analysis focused on the effect of constant temperature on mosquito-pathogen traits and WNV transmission suitability. While our results can be used to gain important insights into potential shifts in the distribution and seasonality of WNV under climate change, the *Culex* life cycle and transmission of WNV depend on numerous other factors, including precipitation, humidity, land cover, and socio-demographic variables, as well as host abundance, diversity, phenology, and immunity, amongst others [17]. Our approach does not consider the relative importance of temperatures’ impact on mosquito-pathogen traits compared to these factors, all of which are potentially important to consider in models of WNV transmission. An extended modelling approach could, for example, allow to predict absolute $R_{0}$ values. This would require considering extending the mosquito-pathogen trait models beyond temperature and incorporating the temperature-independent parameters that cancel out when deriving the relative model. The $R_{0}^{\text{rel}}$ model identifies a transmission risk space that predicts the temperature optimum and limits (where $R_{0}=0$) but lacks the power of an absolute $R_{0}$, which can be used to consider epidemic thresholds to pinpoint outbreak-prone areas and seasons (where $R_{0}>1$). A first step for extending the mosquito-pathogen trait models would be to generate extensive trait measurement data using multifactorial experiments. Bayesian hierarchical models could then be used to fit response surfaces that show how trait performance varies across temperature and additional interacting factors such as humidity [113]. These trait performance surfaces could then be incorporated into transmission dynamic and suitability models to allow better predictions of transmission risk in time and space.

## Conclusion

Using a new Bayesian hierarchical statistical framework for analysing experimental mosquito-pathogen trait measurements, we find that WNV transmission suitability peaks around 24°C with relatively little differences between *Culex* species whereas estimates for temperature limits of transmission are more uncertain. Our novel estimates can inform assessments of the impacts of climate change on WNV transmission. Additional experimental studies are needed on the traits identified as primary contributors to uncertainty in the estimates of temperature limits, temperature optima, and the total uncertainty in transmission suitability, i.e., adult mosquito lifespan, biting rate, and egg viability. More refined data from vector competence studies are also needed to relax some of the more limiting and simplifying model assumptions. To increase the value of experiments for mathematical modelling, studies should ideally test multiple temperatures over a wide range of values. Furthermore, open sharing of raw data would provide the possibility to use the most suitable statistical approach for estimating each trait. Future studies could extend our analyses to more mosquito species, other pathogens, consider other and more detailed hierarchies in the statistical model, or extend our models to additional factors beyond temperature.

## Supporting information

S1 TextSI1, Details on functions fitted to mosquito-pathogen traits. SI2, Summary of mosquito-pathogen temperature response fits. SI3, Formal mathematical description of Bayesian hierarchical models and (hyper)prior specifications. SI4, Population-level trait estimates and sensitivity to between-experiment variability. SI5, Importance of accounting for between-experiment variability. SI6, Taxonomic considerations. SI7, Alternative mosquito abundance models. SI8, Key changes applied to original dataset. SI9, Details on literature search and overview of collected data. SI10, MCMC diagnostics. SI11, Sensitivity and uncertainty analysis. SI12, Comparison to previous $R_{0}^{\text{rel}}\left(T\right)$ estimates.(PDF)
